# Debris extrusion and foraminal deformation produced by reciprocating instruments made of thermally treated NiTi wires

**DOI:** 10.1590/1678-7757-2017-0215

**Published:** 2018-01-16

**Authors:** Myrna Maria Arcanjo Frota, Ricardo Affonso Bernardes, Rodrigo Ricci Vivan, Nilton Vivacqua-Gomes, Marco Antonio Hungaro Duarte, Bruno Carvalho de Vasconcelos

**Affiliations:** 1 Universidade Federal do Ceará, Programa de Pós-Graduação em Odontologia, Fortaleza, Ceará, Brasil; 2 Associação Brasileira de Odontologia, Taguatinga, Distrito Federal, Brasil; 3 Universidade de São Paulo, Faculdade de Odontologia de Bauru, Departamento de Dentística, Endodontia e Materiais Odontológicos, Bauru, São Paulo, Brasil; 4 Faculdade de Odontologia São Leopoldo Mandic, Fortaleza, Ceará, Brasil

**Keywords:** Endodontics, Nickel, Titanium, Root canal preparation

## Abstract

**Objective:**

To evaluate the amount of apically extruded debris, percentage of foraminal enlargement and apical foramen (AF) deformation that occurred during root canal preparation with different reciprocation systems: Reciproc, WaveOne (M-Wire), and ProDesign R (Shape Memory Technology Wire) at two different working lengths (WLs): 0.0 and 1.0 mm beyond the AF.

**Material and methods:**

The AF of 120 root canals in 60 mesial roots of mandibular molars were photographed with stereomicroscope and randomly assigned into four groups: manual, Reciproc (REC), WaveOne (WO), and ProDesign R (PDR); subsequently, they were further subdivided according to the WL (*n*=15). Teeth were instrumented, coupled to a dual collecting chamber, and then another photograph of each AF was captured. Extrusion was analysed by determining the weight of extruded debris. Each AF diameter was measured in pre- and post-instrumentation images to determine deformation, which was analysed, and afterwards the final format of AFs was classified (circular/oval/deformed).

**Results:**

We found no significant differences when analysing each system at different WLs. When considering each WL, REC and WO showed highest extrusion values (*P*<.05); for AF enlargement, differences were observed only for WO, when it was used beyond the AF; differences were observed among M-Wire groups beyond the AF (*P*<.05). AF deformation was observed in all groups; PDR showed the lowest AF deformation values at both WLs; M-Wire groups showed 50% strain beyond the AF.

**Conclusion:**

Authors concluded that beyond the apical limit, the alloy and taper are important aspects when considering extrusion and deformation.

## Introduction

Since their introduction by Walia, Brantley, Gerstein^24^ (1988), nickel-titanium (NiTi) alloy endodontic instruments have undergone several changes to produce further improvement in their properties^4^ ^,^ ^13^. Because NiTi is very sensitive to thermal and mechanical treatments, different manufacturing strategies are capable of producing alloys with differentiated superelastic, resistance and memory characteristics^12^. These treatments have provided instruments made of R-phase NiTi (SybronEndo; Orange, CA, USA), M-Wire NiTi (Dentsply-Tulsa Dental; Tulsa, OK, USA and VDW GmbH; Munich, Germany) and CM-Wire NiTi (Coltène-Whaledent; Cuyahoga Falls, OH, USA), which have improved characteristics of resistance to torsional fracture and cyclic fatigue when compared with instruments manufactured with conventional NiTi alloy^3^ ^,^ ^8^ ^,^ ^12^ ^,^ ^22^. These characteristics supposedly promote the efficiency of chemical-mechanical preparation and reduce the risk of fracture and iatrogenic errors.

Parallel developments of NiTi alloys and different kinematics have been proposed as a way of providing safer, simpler and faster preparation. The reciprocating motion proposed by Yared^26^ is one of the most successful examples in this regard. The kinematics associated with the use of M-Wire instruments, such as Reciproc (VDW GmbH) and WaveOne (Dentsply-Maillefer; Ballaigues, Switzerland), have allowed safe, quick and efficient root canal preparations with a single instrument^12^ ^,^ ^13^ ^,^ ^21^ ^,^ ^26^.

The Reciproc and WaveOne instruments have similar DO and taper: #25 and 0.08, respectively. However, the cross-sections are different, which favour their singular characteristics of resistance to torsional and flexural fracture. As they have a larger metallic mass, the WaveOne (triple-helix cross-section and hollow triangle) is more resistant to torsion; and, in turn, the Reciproc ("S" shaped cross-section) is more flexible^8^. This information reinforces the need for thorough knowledge of the instruments to enable the best indication of the systems in different clinical conditions.

Another clinically relevant aspect of these instruments/kinematics is their greater tendency to extrude debris through the apical foramen (AF) during the mechanical preparation of the root canal system, which can lead to postoperative pain^5^ ^,^ ^23^. This finding is not unanimous in the literature; however, it seems to be related to the design of the instruments (larger or smaller area for debris accumulation between the coils) and kinematics (release of the scrapings collected when the direction of movement is reversed)^1^ ^,^ ^3^ ^,^ ^17^ ^,^ ^21^.

Recently, a new reciprocating instrument was developed, the ProDesign R (Easy Dental Equipment; Belo Horizonte, MG, Brazil), manufactured with a NiTi-based shape-memory alloy (SMT-wire), quite similar to CM-wire. This instrument has DO #25 and constant taper (.06) over its active part^9^. Despite the constant external taper, the instrument has a reduced volume along the active part of its core, which, supposedly, increases the capacity for collecting dentin scrapings produced during preparation, as well as flexibility in the intermediate portions^14^. Its cross-section is "S" shaped; however, it is sharper than that of Reciproc, which would give it greater power cutting ability. Unlike other reciprocating systems that use lower angles, the ProDesign R operates in a counterclockwise rotation of 330° followed by relief rotation of 30° in the opposite direction^14^. Because it is made of SMT-Wire NiTi, it has a controlled memory, which allegedly favours a more centralized root canal preparation^10^. To date, only one study has been available in the literature, presenting promising results related to its bending and cyclic fatigue resistence^16^.

Regarding the definition of the apical limit of instrumentation, Endodontics has been dedicated to investigating possible variations, and understanding the need to disinfect the entire root canal system, not only to a historically predetermined limit (i.e., 1.0 mm short of the AF), but throughout its entire extension, which means right up to the AF. Thus, apical limits considering the root canal length (RCL) of the tooth, or even beyond this, as being an ideal apical stop have appeared in the literature^17^ ^,^ ^19^ ^,^ ^25^. Although not consolidated, this practice could allow irrigation in the apical region and promote a more efficient mechanical debridement of the apical portion, including the AF, optimizing the disinfection of the root canal and favouring its repair^14^ ^,^ ^19^.

However, a major concern about extending the apical limits (i.e., beyond AF) is the possibility that larger quantities of debris, bacteria and irrigators could be extruded through the AF compared with those that could occur during conventional instrumentation^1^ ^,^ ^17^ ^,^ ^20^. This extrusion has commonly been associated with postoperative pain and/or delay in periapical repair^6^. Furthermore, this method could cause possible changes to the format of the AFs due to the limitation of the flexibility of the instruments available at present^6^. Thus, this study evaluated the apical extrusion produced by NiTi instruments manufactured with M-Wire (Reciproc and WaveOne) and SMT-Wire (ProDesign R) alloy, correlating it to the percentage of foraminal enlargement and AF deformation during root canal preparations at two different apical limits (WL1 = 0.0 mm; WL2=1.0 mm beyond the AF). Null hypotheses tested were (1) there would be no differences between instruments regarding apical extrusion of debris, and AF expansion and deformation; and (2) that the WL would have no influence on the apical extrusion of debris, and AF expansion and deformation.

## Material and methods

The sample calculation was performed with the G*Power v. 3.1 for Mac program (Heinrich Heine; Universität Düsseldorf, Düsseldorf, Germany) by selecting the Wilcoxon-Mann-Whitney t-test. We considered data of a previous study, which used unirradicular teeth^17^, thus establishing effect size in this study (1.03). The alpha type error of .05, a beta power of .80 and a ratio N2/N1 of 1 were also stipulated. Thirteen samples *per* group were indicated as the ideal size required for noting significant differences. Because of the risk of tooth loss during the chemical-mechanical preparation, we stipulated a sample of fifteen canals *per* group.

After approval by the Local Ethics Committee (Protocol #1.935.069), a total of 120 root canals in type IV Vertucci mesial roots – only those with slight curvatures (10 to 25°) – of 60 mandibular molars were included in this study. We replaced root canals in which foraminal patency was not possible or with AF diameter greater than 200 μm.

After standard coronal access (#1012 and #3081, KG Sorensen; São Paulo, SP, Brazil), K-type files (Dentsply-Maillefer) were inserted into the root canals until their tips were viewed throughout the AF with the aid of a clinical microscope (Alliance; São Paulo, SP, Brazil) at 16x magnification. When foraminal patency was not achieved, another trial was performed with C-Pilot files #10 (VDW GmbH). In the patent canals, with files placed in the AFs, the RCLs were recorded and digital periapical radiographs (FIT – Digital Radiograph Sensor, Micro Imagem; Indaiatuba, SP, Brazil) were taken to determine the curvatures according to the Schneider^15^ methodology. The occlusal portions of the teeth were also adjusted (sanded) by dental wear to standardize the WLs (20.0±1.0 mm).

Teeth were then placed in a silicone mould (3D, Angelus; Londrina, PR, Brazil) and taken to the stereomicroscope (Stemi 2000C, Carl Zeiss; Jena, Germany) to capture the initial digital photographs of their AFs with AxioVision 4.8 software (Carl Zeiss) using 40× magnification. These images were analysed with Image J software (National Institutes of Health; Bethesda, MD, USA) in which the initial area of AFs (A0) was determined. To enable analysis of apical debris extrusion, instrumentation of the root canals was performed with teeth connected to a container model with a dual chamber^3^, so that each canal had its own collection vial. Prior to fitting the model, the collector vial was carefully weighed (W0) on a precision scale (0.0001 g) (AUW 320, Shimadzu Corp.; Tokyo, Japan) to determine the amount of extruded material; weights were measured in triplicate.

Irrespective of the group or subgroup, distilled water was used as the irrigating solution inserted into the canals through a 5.0 mL plastic syringe (Ultradent Products; South Jordan, UT, USA) with a 30-gauge needle (NaviTip, Ultradent Products). A penetration depth of 5.0 mm short of the WL established and a dynamic irrigation procedure were performed with particular attention to avoid needle locking onto the root canal walls. After each complete removal of the instrument or drill, irrigation associated with a K-file was performed to recap the foraminal patency. Considering variation in the number of pecking movements of instruments/instrumentations, a total volume of 10 mL irrigation solution was proportionally distributed during the preparation procedures.

Two subgroups were established for each group, with the sole purpose of varying the WL: WL1=0.0 mm from RCL (i.e., at the AF) and WL2=RCL+1.0 mm. To eliminate possible heterogeneity in the samples of the four groups and their two subgroups (n=15), teeth were randomly divided between the groups. A single experienced operator performed the preparations according to the following sequence:

### G1.1 and G1.2 – Manual groups (used as controls)

The preparation of the cervical and middle thirds was performed with Gates-Glidden drills #4, #3, and #2 used in descending order until the lowest reached two-thirds of WL or reached the bend of the canal. Instrumentation of the apical third was carried out with hand K-Flexofile (#50 – #25, Dentsply-Maillefer), using instrument #25 as apical file; instruments were used with balanced force motion.

### G2.1 and G2.2 – Reciproc groups

Reciproc R25 instruments driven by motor VDW Silver (VDW GmbH) in "Reciproc All" function were used with gentle in-and-out movements (pecking); the range of motion was limited to 3.0 mm. After each sequence of three pecks, the instrument was completely removed from the root canal and cleaned with gauze.

### G3.1 and G3.2 – WaveOne groups

In this group of root canals, preparation was performed similarly to that described for the Reciproc groups; however, we used the electric motor function "WaveOne All".

### G4.1 and G4.2 – ProDesign R groups

This group of root canals was prepared in a manner similar to that described for the other reciprocating files; however, we used the electric Endo Easy SI motor (Easy Dental Equipment) in the "ProDesign R" function.

Irrespective of the group, canals received final irrigation with 2.0 mL of irrigating solution. Then the tooth set/cover was removed, and the apical portion of the mesial root was gently irrigated with 5.0 mL of distilled water to remove any debris sticking to the external surface. Vials were collected and taken to a dry-heat oven where they were kept at 140°C for 5 h to evaporate their liquid contents. They were again weighed in triplicate (W1), which determined the weight of the material extruded during the root canal system preparation (Ex=W1–W0).

Simultaneously to determining the weight of the extruded contents, teeth were once again assembled in silicone moulds and photographed with a stereomicroscope to verify the final area of AFs (A1). The percentage of foraminal enlargement (PFE) produced by instrumentation was determined by the difference between initial and final areas (PFE=A1–A0). This last image captured was also analysed to determine the final shapes of foramens; for this purpose, the largest and smallest diameters of the AF were assessed. Because of the difference between these diameters, AFs were classified as circular (<.02 mm), oval (>.02 mm and <.06 mm) or deformed (>.06 mm), as adapted from Marroquim and Al-Sayed^11^ (2007).

Data obtained for each of the parameters evaluated were submitted to a normality test that attested the non-normal distribution of values (Shapiro-Wilks). The Mann-Whitney test was used to compare each technique in the two WLs; and Kruskal-Wallis and Dunn tests were used for comparisons between the techniques in each WL; for the foraminal deformation analysis, the Chi-square test was applied. For all tests the level of significance was set at *P*<.05.

## Results

Table 1 shows the median values and range (minimum and maximum) of apical extrusion values for the different preparation techniques found with the two apical limits tested. No statistically significant differences were found when comparing the values attained by each technique for the two apical limits (0.0 mm and +1.0 mm) (*P*>.05). However, when techniques were compared at the same limit, ProDesign R produced significantly lower apical extrusion values in comparison with the other reciprocating techniques (*P*<.05), and with values similar to those of the control (*P*>.05).

**Table 1:** Median (range) of apical extruded debris (grams) produced by the root canal

Group	Working length			
	0.0 mm		+1.0 mm	
Manual	0.0021^a^ ^AB^	(0.0003–0.0046)	0.0031^a^ ^AB^	(0.0011–0.004)
Reciproc	0.0033^a^ ^B^	(0.0024–0.0043)	0.0036^a^ ^B^	(0.0028–0.0052)
WaveOne	0.0031^a^ ^B^	(0.0018–0.0041)	0.0039^a^ ^B^	(0.0029–0.0042)
ProDesign R	0.0016^a^ ^A^	(0.0005–0.0024)	0.0018^a^ ^A^	(0.0004–0.0028)

a,b Different superscript lowercase letters indicate statistically significant differences according to the Mann-Whitney test (*P*<.05), considering each preparation technique.

A,B Different superscript uppercase letters indicate statistically significant differences according to the Kruskal-Wallis and Dunn tests (*P*<.05), considering each working length level.

The median values and range (minimum and maximum) of the percentage of foraminal enlargement produced by the instrumentation techniques are presented in Table 2. With the exception of the groups that used the WaveOne Primary (G3), in which the expansion beyond the AF reached 358.8%, no other technique showed significant differences between the two subgroups, i.e., the same system in the two WLs (*P*>.05). When we analysed the percentage of enlargement produced with the apical limit in the AF, we found no differences between techniques; the control group (manual) had the lowest percentages of enlargement. However, considering the preparation beyond the AF, significant differences were observed between control and G2 and G3 (*P*<.05); there were no differences between G4 and the other groups studied.

**Table 2:** Median (range) percentage increase of apical foramen diameter produced by the root canal preparation techniques at different working lengths

Group	Working length			
	0.0 mm		+ 1.0 mm	
Manual	30.93^a^ ^A^	(14.75–98.46)	73.02^a^ ^A^	(56.12–98.39)
Reciproc	145.2^a^ ^A^	(56.53–187.66)	201.11^a^ ^B^	(120.03–250.72)
WaveOne	121.29^a^ ^A^	(33.79–169.21)	358.80^b^ ^B^	(298.08–450.34)
ProDesign R	47.48^a^ ^A^	(27.99–93.42)	96.14^a^ ^AB^	(58.72–187.21)

a,b Different superscript lowercase letters indicate statistically significant differences according to the Mann-Whitney test (*P*<.05), considering each preparation technique.

A,B Different superscript uppercase letters indicate statistically significant differences according to the Kruskal-Wallis and Dunn tests (*P*<.05), considering each working length level.

**Figure 1:**
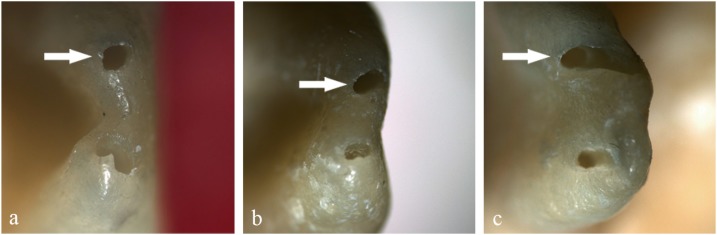
Apical foramen configuration examples after mechanical preparation using different techniques and working lengths (a- circular, b- oval, c- deformed)

**Figure 2:**
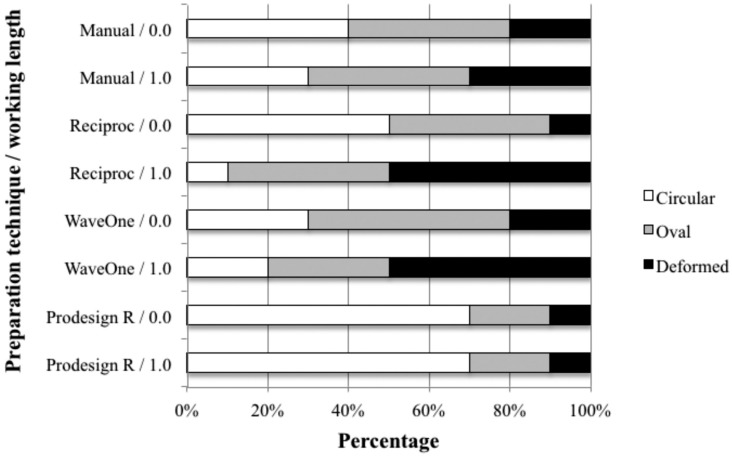
Graphical representation of the occurrence of apical foramen configurations after mechanical preparation using different techniques and working lengths

Figure 1 presents post-preparation foraminal aspects produced and illustrates the classification performed. While the statistical analysis did not show significant differences, findings suggest the influence of the WL used when considering the instruments made of M-Wire (Reciproc and WaveOne) in which the incidence of deformed foramens when the canals were prepared beyond the AF (RCL+1.0 mm) reached 50%. The same behaviour was not observed in teeth prepared with the instrument made of SMT-Wire, in which the percentages of deformed AFs were equivalent in the two WLs (10%); this group had even lower values than those of the manual preparation (20%). Even when used at the level of the AF (0.0 mm), M-Wire NiTi alloy instruments had only 50% (G2) and 30% (G3) of circular AFs; the SMT-Wire instrument showed 70% of circular AFs. Figure 2 shows the compilation of the data obtained in the evaluation of foraminal deformation.

## Discussion

Null hypotheses tested were both partially rejected, since we observed significant differences in apical debris extrusion and foraminal enlargements. However, for foraminal deformation, these differences were not statistically significant.

To understand the behaviour of apical extrusion produced by the instrument, we determined the percentage of foraminal enlargement and classification of these AFs regarding their formats. In addition, this study evaluated the possible influence on the patterns of these findings, in relation to two apical instrumentation limits, AF and beyond it (WL2=RCL+1.0 mm). To date, no study has evaluated the extrusion produced by any reciprocating instrument made of SMT-Wire NiTi-based alloy. Similarly, there were no references in the literature to final foraminal design associated with apical extrusion or in preparations made up to the AF or beyond it.

Mesial root canals of mandibular molars with slight curvatures and AF patents with diameters lower than 200 μm were used. This configuration was intended to approximate the conditions of the study to clinical reality, without risking the homogeneity of the sample. Studies with more constricted root canals or larger curvatures may find results differing from those observed here. The dual chamber model used here is enshrined in literature and has been used by several studies that evaluated the extrusion produced by various preparation techniques, either by extrusion of debris^1^ ^,^ ^3^ ^,^ ^17^ ^,^ ^18^ ^,^ ^22^ or bacteria^20^ ^,^ ^21^. Similarly, observation of the shape of AFs by recording their format in photographic documentation has also been used in the literature^6^ ^,^ ^11^; however, to this day, only two classifications have been used (circular and oval). The authors of this study decided to include the deformed profile according to the findings that suggested that this form was an occurrence commonly observed in some groups.

Results suggest a greater influence of the NiTi-based alloy type regarding the extrusion of debris and foraminal format produced by reciprocating systems. Concerning the enlargement of AF, the apical limit seemed to have had a more intense influence, possibly due to the increased metal mass of the instrument, depending on the taper, to surpass the AF. However, the overall analysis of the observations made here has helped to gain better understanding of the findings.

The Reciproc and WaveOne instruments caused the highest apical extrusion values, with no difference between them. This similarity has previously been pointed out by De Deus, et al.^3^ (2015) and Topçuoğlu, et al.^22^ (2016). Silva, et al.^17^ (2016) evaluated the apical extrusion promoted by Reciproc instruments in two working lengths, at the AF and 1.0 mm shorter; however, there was no information about their behaviour in lengths beyond the AF threshold at which this similarity was also observed. Likewise, to this day, no comparisons of these instruments with those made of SMT-Wire were found in the literature. Nevertheless, they were also systems that produced the highest values of foramen enlargement and percentage of deformed foramen, irrespective of the WL considered, which could possibly explain why they extruded more debris. Due to having less metal mass and thus more flexibility, it could be considered that Reciproc instruments should provide less deformation; however, this feature seems to have been offset by their greater cutting power, thereby producing foraminal distortions equivalent to those of the WaveOne instrument. It must be considered that the research method used here obliged the use of distilled water as irrigant, therefore, the clinical results of the use of sodium hypochlorite, or even a less irritant solution as irrigant, could be strongly considered, mainly in over-extended WLs.

Another factor was the difference in the taper of the ProDesign R in comparison with WaveOne and Reciproc instruments. The ProDesign R has a .06 taper, consequently less metallic mass than the WaveOne and Reciproc systems that have a .08 taper. Results observed for the ProDesign R instrument have no parallel in the literature; however, considering the comparison with other reciprocating instruments, the authors could state that since it produced the best results, it was a feasible option for use during root canal system preparation. Not only did this instrument offer the best results of foraminal extrusion of debris, it also differed from other mechanized instruments in both WLs, offering similar results to those presented by the manual instrumentation (control). This finding was consistent with the analysis of expansion and foraminal deformation, which showed that this instrument had the lowest percentage of foraminal enlargement – 47.48% (RCL) and 96.14% (RCL+1.0 mm) –, in spite of showing no statistically significant differences in comparison with other mechanized systems. This system also produced less AF deformation – 20% oval and 10% deformed at both WLs. Possibly, these findings could be the remarkable result attributable to the NiTi-based alloy of which the instrument is made; other factors could be related, such as the cross-section, rotation angles and smaller taper of the instrument. Higher foraminal enlargement and deformation values may favour higher values of root filling material extrusion into the apical tissues, which could harm the apical repair process^7^.

Undeniably, it would have been better if this evaluation had been carried out with instruments of similar design (size/taper); however, manufacturers consider that those used in this study are better suited for use in root canals similar to those used in this research. Moreover, all of the files had a #25 tip, the Reciproc and WaveOne had a .08 taper at their tips, and the Reciproc and ProDesign R presented "S" shaped cross-sections, indications and similarities that could justify this evaluation. Furthermore, it should be understood that the NiTi-based alloy, of which the instruments are manufactured, should not be considered the only factor to justify the results, because it would be difficult to separate the alloy from the design of the files.

According to results, the authors observed that performing chemical-mechanical preparation with the WL established in the AF and beyond it could produce deleterious changes to original anatomy and may compromise the success of endodontic treatment, as mentioned by Çapar, et al.^2^ (2015). However, the introduction of instruments made with shape-memory technology may be an option for preparations performed at the foraminal level. There is no literature discussing the ideal percentage of foraminal enlargement to achieve maximum decontamination of the apical region; however, this study shows that in cases in which the Endodontist, in spite of knowing the risks involved, chose to overextend the preparation in an attempt to increase the AF debridement – or this occurred accidentally during instrumentation – highly flexible instruments, such as those made of SMT-Wire NiTi-based alloy, promoted less foraminal deformation and extrusion of debris.

## Conclusions

Under the conditions of this study, the authors were able to conclude that all the instrumentation systems produced apical debris extrusion and foraminal deformation; however, rather than the apical limit used, the NiTi-based alloy and the taper were the factors that influenced the results of the reciprocating instruments. The ProDesign R system, made with shape-memory technology, and the .06 taper, showed the best results.
